# ERβ and NFκB—Modulators of Zearalenone-Induced Oxidative Stress in Human Prostate Cancer Cells

**DOI:** 10.3390/toxins12030199

**Published:** 2020-03-22

**Authors:** Karolina Kowalska, Dominika Ewa Habrowska-Górczyńska, Kamila Domińska, Kinga Anna Urbanek, Agnieszka Wanda Piastowska-Ciesielska

**Affiliations:** 1 Medical University of Lodz, Department of Cell Cultures and Genomic Analysis, Zeligowskiego 7/9, 90-752 Lodz, Poland; dominika.habrowska@umed.lodz.pl (D.E.H.-G.); kinga.urbanek@umed.lodz.pl (K.A.U.); agnieszka.piastowska@umed.lodz.pl (A.W.P.-C.); 2 Medical University of Lodz, Department of Comparative Endocrinology, Zeligowskiego 7/9, 90-752 Lodz, Poland; kamila.dominska@umed.lodz.pl

**Keywords:** zearalenone, prostate, NFKB, estrogen receptor β, oxidative stress, mycotoxins

## Abstract

Nuclear factor kappa-light-chain-enhancer of activated B cells (NFκB) is commonly expressed in prostate cancer (PCa) cells and is associated with increased proliferation, metastases and androgen independence. Zearalenone (ZEA) is one of the most common mycotoxins contaminating food, which might mimic estrogens and bind to estrogen receptors (ERs). The ratio of androgens to estrogens in men decreases physiologically with age, and is believed to participate in prostate carcinogenesis. In this study, we evaluated the role of NFκB and ERβ in the induction of oxidative stress in human PCa cells by ZEA. As observed, ZEA at a dose of 30 µM induces oxidative stress in PCa cells associated with DNA damage and G2/M cell cycle arrest. We also observed that the inhibition of ERβ and NFΚB via specific inhibitors (PHTPP and BAY 117082) significantly increased ZEA-induced oxidative stress, although the mechanism seems to be different for androgen-dependent and androgen-independent cells. Based on our findings, it is possible that the activation of ERβ and NFΚB in PCa might protect cancer cells from ZEA-induced oxidative stress. We therefore shed new light on the mechanism of ZEA toxicity in human cells.

## 1. Introduction

Oxidative stress in cancer cells plays a dual role: the generation of reactive oxygen species (ROS) in cells increases the proliferation and migration of cells and participates in the initiation and progression of PCa [1], but on the other hand, excessive ROS production induces DNA damage and triggers apoptosis [2]. Nuclear factor kappa-light-chain-enhancer of activated B cells (NFΚB) signaling pathway is believed to be a link between inflammation and cancer, due to the induction of proliferation, blocking apoptosis in cells and maintaining angiogenesis [3], and the induction of oxidative stress with hypoxia inducible factor 1 alpha (HIF-1α) and estrogen receptor β (ERβ) [4]. The constituent activation of NFΚB is reported in androgen-independent PCa cells, although binding sites for NFΚB are found on androgen receptor (AR) promoters [5].

Zearalenone (ZEA) is reported to be one of the most common mycotoxins present in everyday diets. Based on its structural similarity to estrogen, ZEA is able to directly bind to estrogen receptors (ERs) in cells and affect hormonal regulation in humans [6]. In recent years, a lot of research has been conducted to understand the molecular mechanism of toxicity of ZEA and assess its effects on human health [7]. So far, ZEA is considered noncancerogenic to humans; nevertheless, research has reported that it might have a carcinogenic effect via the induction of proliferation, migration and invasion of cells [8]. In contrast, ZEA is also reported to induce oxidative stress, DNA damage, apoptosis and autophagy [9]. The sensitivity of cells seems to be different; porcine cells are believed to be the most sensitive to ZEA-induced toxicity [10]. ZEA is reported to modulate the activation of inflammasome via p65 in INS-1 cells [11]; nevertheless, a direct association between ZEA and NFΚB has not been found yet, especially in the case of cancer cells, where the activation of NFΚB plays a role.

Our previous results showed that ZEA might have a two-fold role in PCa cells, inducing both the proliferation and migration of cells, as well as the death of PC3 cells. It is known that estrogen receptor α (ERα) and ERβ play opposite roles in the progression of PCa [6]. In that case, we postulate that ZEA via binding to different ERs might trigger a contradictory effect in cells, and we decided to verify this hypothesis. Mak et al. reported that ERβ in PCa regulates the expression of NFΚB via HIF-1α [4], whereas our observations showed that ZEA influences the expression of *HIF-1α* [12]. Thus, it is probable that both ERβ and NFΚB might play a role in ZEA-induced oxidative stress. Therefore, we decided firstly to evaluate whether ZEA induces oxidative stress in PCa cells, in both androgen-dependent and androgen-independent PCa cell lines reported to express ERβ and lacking ERα [13]. An inhibitor of NFΚB (BAY 117082) and a specific antagonist of ERβ, i.e., 2-Phenyl-3-(4-hydroxyphenyl)-5,7-bis(trifluoromethyl)-pyrazolo [1,5-α]pyrimidine (PHTPP), were used to study the role of ERβ and NFΚB in ZEA-induced oxidative stress.

## 2. Results

### 2.1. The Effect of ZEA on PCa Cell Viability

**Figure 1:**
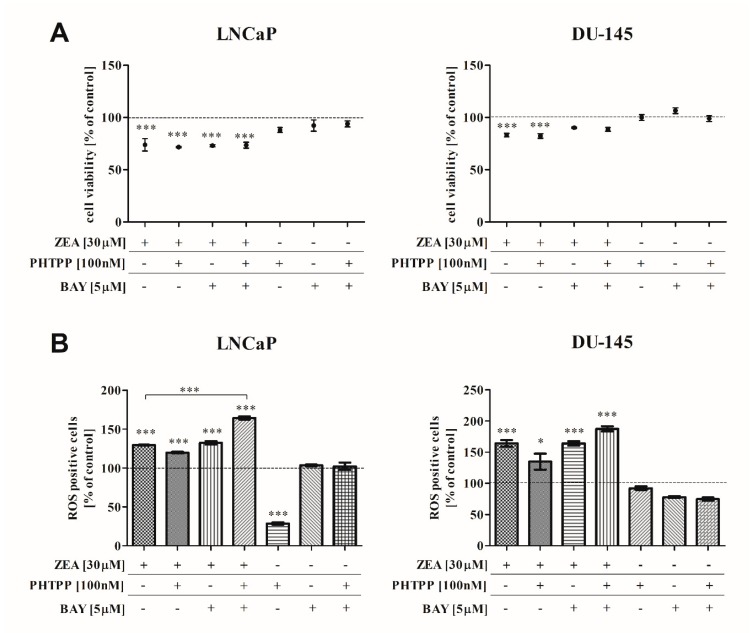
(**A**) Viability of cells after ZEA and/or ERβ and NFΚB inhibitors treatment. Cell viability was determined with MTT reagent after 48 h of exposure. (**B**) Induction of oxidative stress after ZEA treatment in PCa cells. The number of ROS positive cells was determined using a Muse Cell Analyzer. The results are expressed as a percentage of control. Significant differences were calculated with one-way ANOVA with Bonferroni post hoc test and expressed as mean ± SE. * *p* < 0.05, *** *p* < 0.001. Asterisks above bars indicate significance compared to the control. ZEA—zearalenone, PHTPP—ERβ inhibitor, BAY—NFΚB inhibitor, Cnt—control.

To assess the inhibitory effect induced by ZEA and the potential influence of the ERβ and NFΚB pathways, we evaluated whether ZEA itself and in combination with PHTPP and BAY decreases the viability of PCa cells. The results are shown in Figure 1A. We observed that in all cell lines, treatment with ZEA significantly decreased cell viability compared to control cells (*** *p* < 0.001). No changes were observed after adding PHTPP and/or BAY. The sensitivity of prostate cancer cells to ZEA-induced cell death was different: androgen-independent DU-145 seems to be less sensitive compared to LNCaP cells.

### 2.2. ZEA-Induced DNA Damage and ROS Production

To determine whether NFΚB and ERβ might participate in the ZEA-induced DNA damage and ROS production, NFΚB and ERβ inhibitors were used. Although the observed decrease in cell viability was not so high, in all tested PCa cell lines, a significant increase in the number of ROS positive cells was observed after treatment with ZEA and ZEA + inhibitors (Figure 1B). Although DU-145 cells seems to be less sensitive to ZEA based on viability results, a higher number of ROS positive cells was observed. The simultaneous inhibition of ERβ and NFΚB increased ZEA-induced oxidative stress, and significant results were observed for LNCaP cells (*** *p* < 0.001). We observed a significantly higher number of ROS positive cells after ZEA + BAY + PHTPP treatment, compared to cells treated only with inhibitors (*** *p* < 0.001). Interestingly, we also observed that the addition of PHTPP to LNCaP cells caused a significant decrease in the number of ROS positive cells, compared to the control (*** *p* < 0.001).

**Figure 2:**
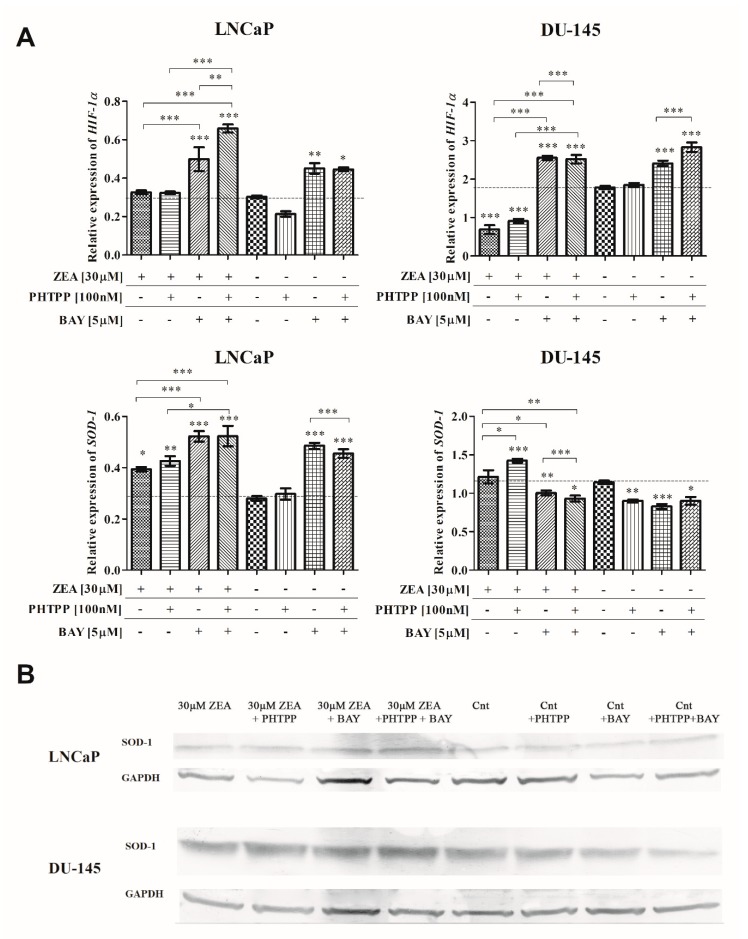
(**A**) The expression of *HIF-1α* and *SOD-1* after ZEA and/or ERβ and NFΚB inhibitors treatment. The expression was evaluated with RT-qPCR. The results are presented as relative expression. One-way ANOVA with Bonferroni post hoc test was used to evaluate the results. *p* < 0.05 was considered as significant. * *p* < 0.05, ** *p* < 0.01, *** *p* < 0.001. Asterisks above bars indicate significance compared to the control. (**B**) Representative results of Western blot analysis of SOD-1 expression. ZEA—zearalenone, PHTPP—ERβ inhibitor, BAY—NFΚB inhibitor.

Next, the expression of *HIF-1α* and *SOD-1* was evaluated. In LNCaP cells, neither ZEA nor ZEA + PHTPP treatment caused any significant change in *HIF-1α* expression (Figure 2). *SOD-1* expression was significantly increased after ZEA and ZEA + PHTPP treatment (* *p* < 0.05, **p < 0.01, respectively). The expression of both genes was increased after simultaneous treatment with ZEA and both inhibitors (*** *p* < 0.001), compared to ZEA treatment alone. A different change of the expression of *HIF-1α* and *SOD-1* was observed in DU-145 cells. ZEA and ZEA + PHTPP treatment caused a significant decrease in *HIF-1α* expression (*** *p* < 0.001), but similarly to LNCaP cells, the addition of BAY caused an increase in the expression compared to ZEA and ZEA + PHTPP treatments (*** *p* < 0.001). In both cells lines, the addition of BAY to control cells caused an increase in *HIF*-1α expression. The increase in the expression of *SOD-1* caused by ZEA and ZEA + PHTPP was also observed in DU-145 cells; however, in contrast to LNCaP cells, the addition of BAY to ZEA-treated cells caused a significant decrease in *SOD-1* expression. A similar decrease was observed after adding BAY to control cells (***p < 0.001 and *p < 0.05, respectively). On the protein level, the changes were only slight in the case of LNCaP cells (Table 1), but the decrease of its expression was visible for ZEA treatment. The observed changes in expression of SOD-1 in DU-145 cells were different, as observed in the mRNA level. Treatment with ZEA caused a decrease in SOD-1 expression, compared to nontreated cells. The addition of PHTPP increased the expression of SOD-1. In the case of BAY treatment, treatment with ZEA + BAY caused a decrease in expression which was greater than that caused only by ZEA treatment. The increase of SOD-1 expression was observed only for ZEA + PHTPP + BAY treatment, compared to the control. Similar to the mRNA results, the addition of BAY to control cells decreased the expression of SOD-1.

**Figure 3:**
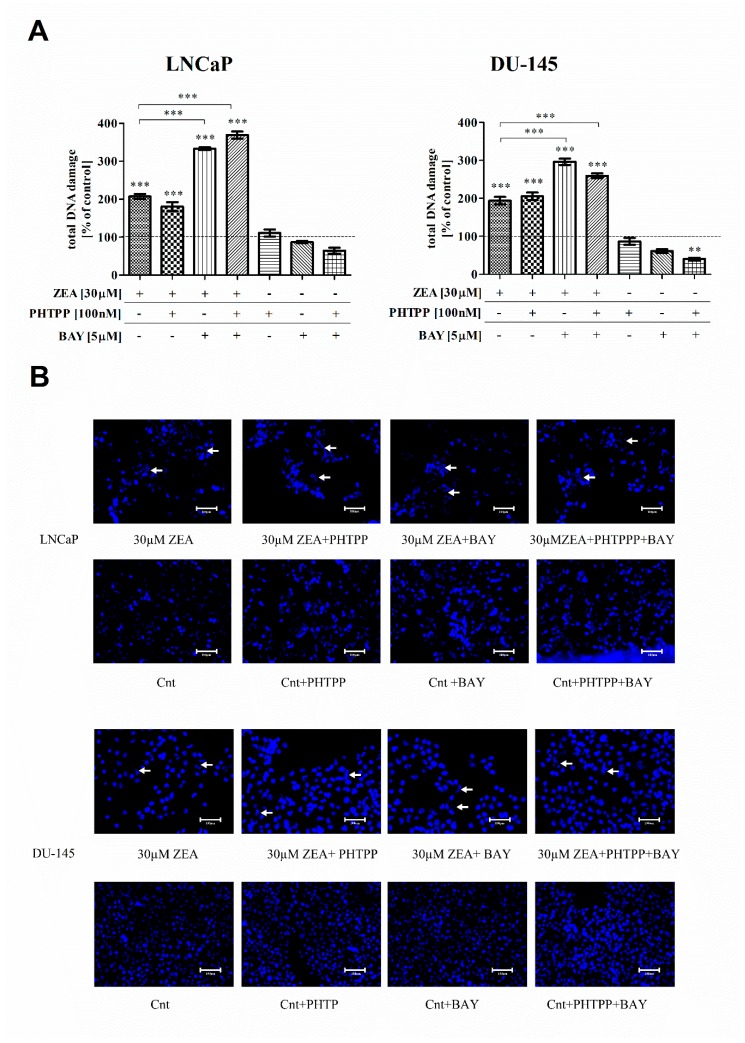
Lack of NFΚB increases ZEA-induced DNA damage in prostate cancer cells. (**A**) DNA damage was counted on Muse Cell Analyzer and presented as % of control cells. Significance of the results was calculated with one-way ANOVA with Bonferroni post hoc test. *p* < 0.05 indicated statistical significance. ** *p* < 0.01, *** *p* < 0.001. Asterisks above bars indicate significance compared to the control. (**B**) DAPI staining of the nuclei of cells. Representative cells with fragmented DNA are marked with white arrows, optical magnification 460×. ZEA—zearalenone, PHTPP—ERβ inhibitor, BAY—NFΚB inhibitor.

Oxidative stress in cells is associated with DNA damage. Consequently, we decided to verify the DNA damage in PCa cell lines treated with ZEA. The detection of activated ATM and H2A.X in cells determines DNA double-strand breaks (DNA damage). As shown in Figure 3A, ZEA in the tested dose caused a significant increase in DNA damage compared to the control in both cell lines (** *p* < 0.01, *** *p* < 0.001). Treatment with both ZEA and PHTPP had no significant effect on DNA damage compared to ZEA treatment. In all the tested cell lines, simultaneous treatment with ZEA and BAY caused a higher increase in DNA damage than ZEA alone (** *p* < 0.01, *** *p* < 0.001). Similarly, the addition of both inhibitors increased DNA damage (*** *p* < 0.001).

As shown in Figure 3B, DNA damage was also imprinted in the nuclei of cells. We observed fragmented cell nuclei in all cells treated with ZEA. The number of cells with fragmented DNA was visibly higher after ZEA and inhibitors, compared to ZEA treatment alone.

### 2.3. ZEA-Induced Cell Cycle Arrest in G2/M Cell Cycle Phase

**Figure 4:**
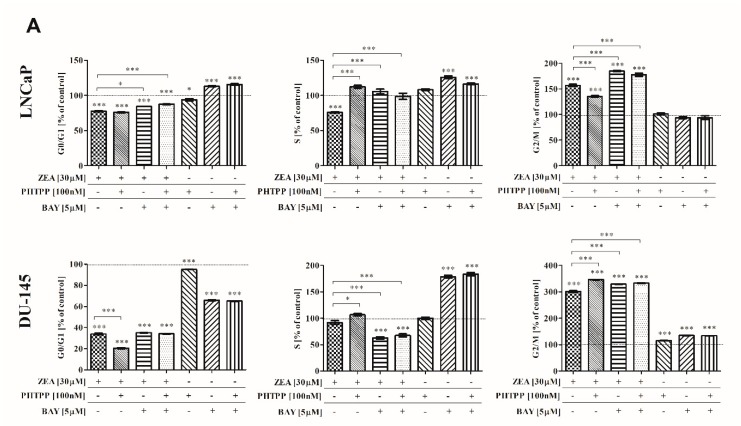

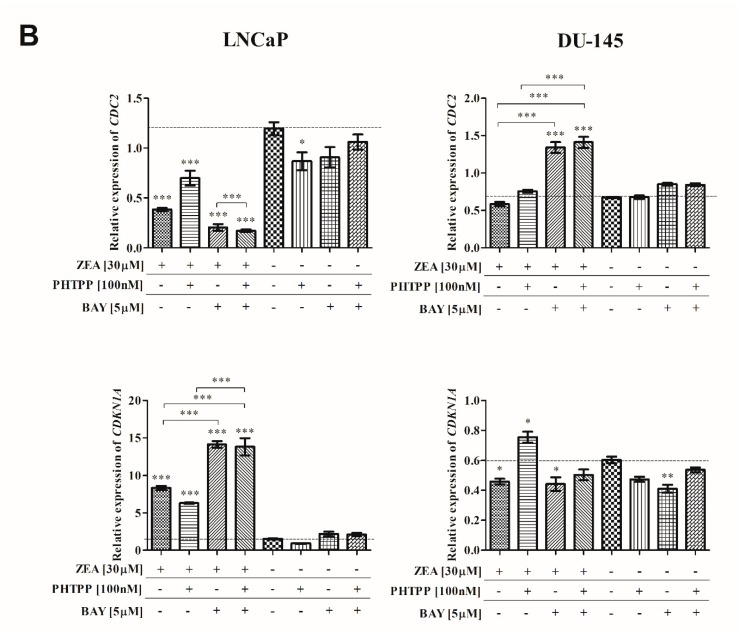
(**A**) Cell cycle evaluation after ZEA and/or ERβ and NFΚB inhibitors treated PCa cells. The distribution of cells was analyzed with Muse Cell Cycle Analysis Kit. The results are expressed as % of control cells. (**B**) The expression of CDC2 and CDKN1A after ZEA and/or ERβ and NFΚB inhibitors treated PCa cells. The expression of genes was evaluated with RT-qPCR method. The results are expressed as a mean ± SE. Statistical significance was calculated with one-way ANOVA with Bonferroni post hoc test (*p* < 0.05). * *p* < 0.05, ** *p* < 0.01, *** *p* < 0.001. Asterisks above bars indicate the significance compared to the control. ZEA—zearalenone, PHTPP—ERβ inhibitor, BAY—NFΚB inhibitor.

To investigate whether the induction of oxidative stress in cells might be associated with changes in cell cycle, we evaluated cell cycle progression. As shown in Figure 4A, in all tested cell lines, we observed that treatment with ZEA caused a statistically significant decrease in the number of cells in the G0/G1 cell cycle phase (*** *p* < 0.001). A statistically significant increase in the number of cells in G2/M cell cycle phase (*** *p* < 0.001) was also observed. In LNCaP and DU-145 cells, the addition of both inhibitors triggered an increased number of cells in the G2/M cell cycle phase (*** *p* < 0.001) The changes in cell cycle progression were also confirmed with the expression of *CDC2* and *CDKN1A* (Figure 4B). A contradictory pattern of *CDC2* expression was present in LNCaP and DU-145 cells, where the expression was decreasing and increasing, respectively, after the addition of inhibitors, compared to ZEA treatment alone. Significant changes in the expression of *CDKN1A* after the addition of inhibitors were observed in androgen-dependent LNCaP cells (*** *p* < 0.001).

### 2.4. Nrf2 and NFΚB in ZEA-Induced Oxidative Stress

**Figure 5:**
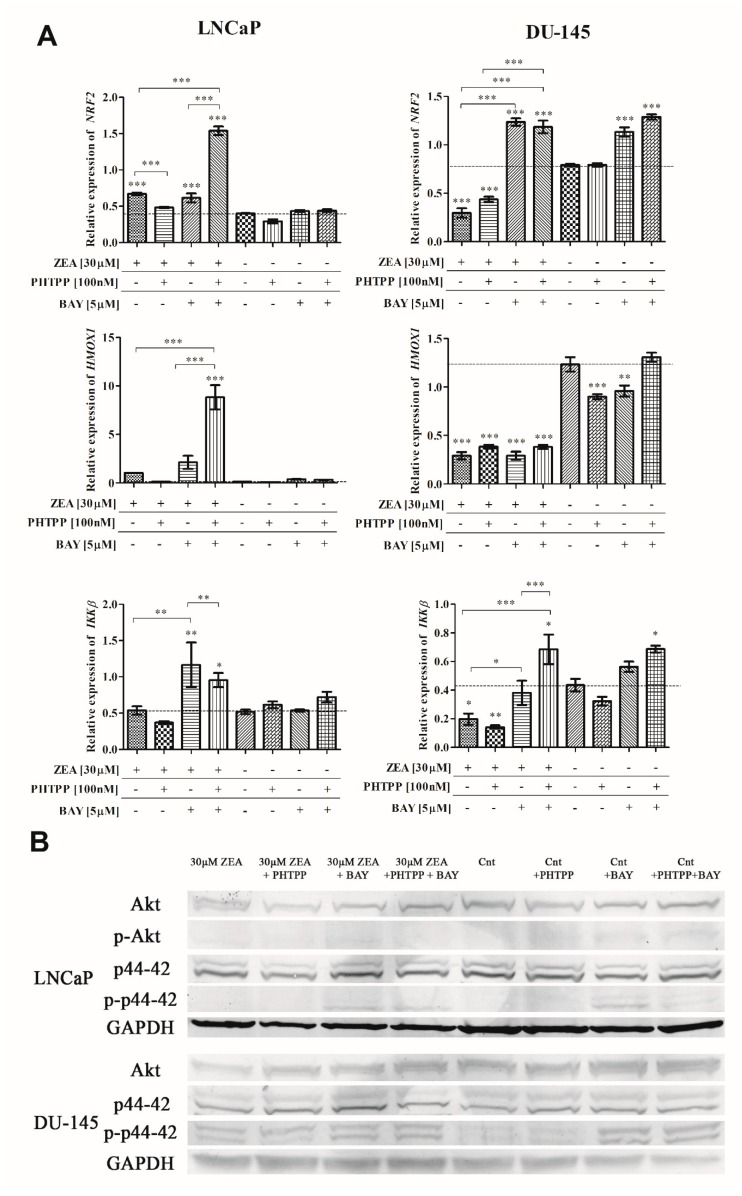
(**A**) ZEA modulates expression of NRF2, HMOX1 and IKKβ in PCa cells. The expression of genes was evaluated in RT-qPCR. The results are presented as mean ± SE. Statistical significance of the results was calculated with one-way ANOVA with Bonferroni post hoc test (*p* < 0.05). * *p* < 0.05, ** *p* < 0.01, *** *p* < 0.001. Asterisks above bars indicates the significance compared to the control. (**B**) The expression of Akt and p44–42 in ZEA treated prostate cancer cells. Representative results of Western blots. ZEA-zearalenone, PHTPP-ERβ inhibitor, BAY- NFΚB inhibitor.

Our previous results showed that ZEA-induced oxidative stress might be associated with changes in the expression of the genes responsible for the oxidative stress response [12]. In that case, the expression of *NRF2*, *HMOX1* and *IKKβ*, due to its documented role in oxidative stress (NFΚB signaling pathway), and ERβ was evaluated (Figure 5A). In LNCaP cells, a significant increase in the expression of *NRF2* was observed after treatment with ZEA; the opposite was observed in DU-145 cells. For LNCaP cells, the addition of both inhibitors to ZEA treated cells caused a significantly elevated expression, compared to ZEA treatment alone (*** *p* < 0.001). In DU-145 cells a significant increase was observed for BAY and BAY + PHTPP addition to ZEA treatment, compared to ZEA alone (*** *p* < 0.001). In LNCaP cells, a similar effect to *NRF2* expression was observed for *HMOX1* expression (*** *p* < 0.001). In DU-145, a significant decrease in the expression of *HMOX1* was observed for all tested cells treated with ZEA (***p < 0.001), but no differences were observed after the addition of inhibitors. Due to the fact that IKKβ was previously reported to participate in the ERβ-NFΚB signaling pathway, we decided to evaluate its expression. An increase in *IKKβ* expression in LNCaP cells after ZEA + BAY and ZEA + BAY + PHTPP treatment (* *p* < 0.05, ** *p* < 0.01) was observed, but it was statistically significant only for BAY addition, compared to ZEA treatment alone (*** *p* < 0.001). In DU-145 cells, the expression of *IKKβ* was decreased after ZEA and ZEA + PHTPP treatment (* *p* < 0.05, ** *p* < 0.01). The addition of the NFΚB inhibitor significantly increased the expression of *IKKβ*, compared to ZEA treatment alone (*** *p* < 0.001). This result indicates that the androgen dependence of PCa cells might cause a different response among well-known antioxidative stress signaling pathways to ZEA.

### 2.5. Akt and p44-42 Expression after ZEA Treatment

The MAPK signaling pathway was previously reported to participate in the effect of ZEA [9]. In that case, the expression of Akt and p44-42 was evaluated (Table 2). Representative results of Western blots are presented in the Figure 5B. The expression of Akt was decreased by ZEA with the exception of treatment with ZEA and both inhibitors in DU-145 cells. A similar effect was also observed for the control of both inhibitors, indicating that blocking ERβ and NFΚB in androgen-independent cells increases the expression of Akt. Phospho-Akt was not detectable in DU-145 cells. In the case of LNCaP cells, the expression of Akt was almost not changed, whereas the expression of phospho-Akt was increased after the addition of inhibitors, compared to control cells. Thus, it is possible that a lack of active ERβ and NFΚB might activate the Akt signaling pathway in PCa cells. The expression of p44-42 was almost unchanged in both cell lines, whereas its phosphorylated form was decreased after the addition of ZEA and increased with the addition of inhibitors for both cell lines.

## 3. Discussion

A lot of studies have reported that ZEA is an antiproliferative, genotoxic and pro-apoptotic agent in in vitro and in vivo models [14]. Nevertheless, there is a limited information about the detailed molecular effect of ZEA on prostate tissue in humans. It is known that ZEA and its active metabolites via their estrogenic properties modulate the production of steroid hormones in males and females [15]. Our previous results showed that ZEA might induce both apoptosis and proliferation in PC3 cells, expressing both ERs [16]. Based on our research, it seems that ERα is necessary for the ZEA-induced proliferation of cells [17], while ERβ might play a contradictory role. In this study, for the first time, we showed that ZEA in high doses induces oxidative stress in PCa cells, which is linked with DNA damage and cell cycle arrest in the G2/M cell cycle phase. Previous studies have also reported that the induction of oxidative stress by ZEA is associated with DNA damage in intestine [18,19], human embryonic stem cells [20] and hepatic cells [21]. The transition from the G2 to M phase of the cell cycle is mainly regulated by a complex of cyclin B1 and cdc2. p21 encoded by the *CDKN1A* gene negatively regulates the complex of cyclin B1 and cdc2 [22]. Although we observed significant changes in the expression of both *CDC2* and *CDKN1A* after ZEA treatment, the changes in the expression pattern were different among two PCa cell lines. This observation might be associated with the fact that these two cell lines represent a different models of PCa with a different differentiation ratio and invasiveness of cells. A cell cycle arrest in the G2/M phase associated with ZEA-induced oxidative stress has also been observed by others [22,23]. The MAPK and PI3K/Akt cell signaling pathways are reported to be involved in ZEA-induced effects in cells [24]. Similar to previous studies, we observed that in PCa cells, ZEA modulates the expression of p-Akt and p-p44/42. An increase in p-Akt expression in LNCaP cells was observed after the addition of inhibitors to ZEA treatment, which correlates with higher ROS production and DNA damage. In DU-145 cells, a similar increase was observed in p-p44–42 expression. The induction of the PI3K/Akt signaling pathway was previously associated both with ZEA-induced autophagy and oxidative stress [25,26]. Although the activation of the PI3K/Akt signaling pathway is mostly associated with the proliferation of cancer cells, similar to others, we observed increased phosphorylation of Akt in cells treated with ZEA + BAY as well as ZEA + BAY + PHTPP in LNCaP cells. A similar anticarcinogenic effect was observed for flavonoid-induced PCa cell death [27].

ERβ is believed to be antiproliferative and pro-apoptotic, although its effect in PCa cells seems to be more sophisticated [27], based on the reports showing that ERβ2 and ERβ5 isoforms play a tumor-promoting role [28]. We previously observed that ERβ in normal prostate epithelial cells plays a protective role in ZEA-induced oxidative stress [12]. We therefore wondered whether a similar effect would also be observed in PCa cells. The results of this study showed that the role of ERβ in ZEA-induced oxidative stress is different, as observed previously [12]. ERβ per se seems to play a nonsignificant role in ZEA-induced oxidative stress, or a significant one in the case of cell cycle regulation in two different PCa cell lines. We observed a significant decrease in the number of LNCaP cells in the G2/M cell cycle phase after blocking ERβ, while the opposite was observed in DU-145. However, the results do not correlate with ROS production and DNA damage. Based on this, we assumed that the role of ERβ in ZEA-induced oxidative stress in PCa cells is different, compared to prostate epithelial cells [12], which provides interesting information for the field of mycotoxin research. On the other hand, we observed that the simultaneous blocking of ERβ and NFΚB increases ZEA-induced oxidative stress in PCa cells. The link between ERβ and NFΚB in PCa cells has been suggested by others [4]. It might be possible that an interplay between ERβ and NFΚB is crucial in the response of cancer cells to ZEA; however, this needs to be confirmed in further studies. It might also be possible that the activation of NFΚB decreases the sensitivity of PCa cells to the induction of oxidative stress. Nevertheless, it should be also highlighted that other estrogen receptors might participate in the effect of ZEA. Interestingly, He at al. observed that the inhibitory effect of ZEA (30 µM) on pig follicle stimulating hormone (FSH) was associated with the nonclassical membrane estrogen receptor GPR30 [29]. The possible involvement of GPR30 has also been suggested by others [30]. Therefore, the possible involvement of GPR30 in ZEA-induced oxidative stress in cancer cells need to be confirmed. It is also possible that the oxidative stress caused by ZEA in PCa cells is directly associated with DNA damage which is caused by non-ERs signaling, similarly to other compounds which are believed to mimic estrogens [31]. Recently, Wang et al. suggested that some of the toxic effects of ZEA cannot be explained by estrogen binding sites, and may be associated with miRNA regulation in TM3 Leydig cells [32].

The blocking of NFΚB signaling in PCa results in the reduction of metastases, invasiveness and angiogenesis. The constitutive activation of NFΚB is associated with poor prognoses [33]. It is also suggested that targeting NFΚB might restore the sensitivity of castration-resistant prostate cancer (CRPC) cells to AR antagonist [34]. Van Uden et al. showed that even in normoxia, NFΚB regulates *HIF-1α* expression [35]. The interplay among NFΚB, Nrf2 and AR signaling pathways in PCa [1] with the modulation of the expression of p65 [12], as we observed, as well as NRF2 in prostate cells after ZEA exposure, indicates the possible involvement of NFΚB signaling in ZEA-induced toxicity. We observed that the blocking of NFΚB resulted in increased ROS production and DNA damage in both PCa cell lines, and was associated with cell cycle arrest in the G2/M cell cycle phase and the modulation of *SOD-1* and *HIF-1α* expression in LNCaP and DU-145 cell lines, although the changes in the expression were different for those two cell lines. In both cell lines, we observed changes in the expression of *NRF2* and *HMOX1*, indicating the activation of an oxidative stress defense mechanism by the Nrf2 signaling pathway. Although androgen-independent PCa cells seem to be less sensitive to ZEA-induced toxicity, the observed increase in ROS positive cells and DNA damage was similar to that of androgen-dependent PCa cells. Differences in the response of these two cell lines were observed previously by Ravenna et al., who reported that NFΚB is the main transcription factor in response to hypoxia and inflammation in DU-145 cells, but that such effects were not present in LNCaP cells [36]. Moreover, the constituent activation of NFΚB was reported to be present in lymph node metastases of PCa [37]. This might also explain the different response of LNCaP cells, due to the fact that LNCaP cells are derived from lymph node metastases of PCa patients, whereas DU-145 are derived from brain metastases. Nevertheless, the observed different patterns in gene expression observed after ZEA treatment indicate that androgen-dependent and -independent PCa cells might have different sensitivities to ZEA. The activation of the Nrf2 signaling pathway after ZEA exposure was observed by Cheng et al. in jejunum of postweaning gilts [38]. A similar effect of ZEA on Nrf2 signaling was also observed by Long et al. in mice Sertoli cells [39]. Our study confirmed previously suggested crosstalk between Nrf2 and NFΚB in PCa cells, and, for the first time, suggested the involvement of NFΚB in ZEA-toxicity. The fact that the highest increase in ROS generation and DNA damage of PCa cells after ZEA exposure was observed for simultaneous treatment with PHTPP and BAY indicates that ERβ might also participate in NFΚB-Nrf2 crosstalk in PCa. The loss of ERβ was associated with inflammation in PCa and increased activation of NFΚB mediated by HIF-1α [4]. Our results showed that active ERβ and NFΚB might protect PCa cells from ZEA-induced oxidative stress, but this statement needs to be confirmed in further studies.

**Figure 6:**
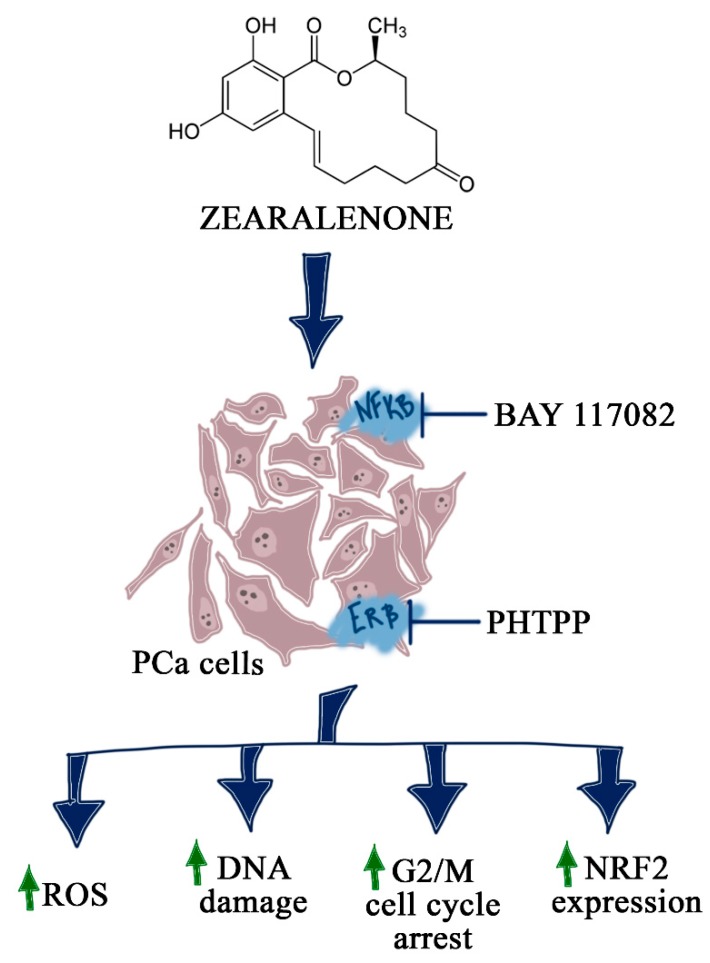
Summarized schema of the results of the study. BAY 117082-NFκB inhibitor, PHTPP—ERβ inhibitor.

Interestingly, beside the different response of DU-145 and LNCaP cells to ZEA, we also observed that the addition of the ERβ inhibitor PHTPP to LNCaP cells significantly reduced oxidative stress, whereas that effect was not observed in DU-145 cells. LNCaP cell growth might be modulated by both androgens and estrogens, as reported previously [40]. ERβ activation is believed to decrease the viability of PCa cells [29]. The observed decrease in ROS-positive cells after blocking ERβ might confirm this hypothesis, but this needs to be studied further. We also observed that the blocking of NFΚB in PCa cells increases the expression of *HIF-1α*, *SOD-1*, and the Nrf-2 pathway, and modulates the number of cells in the G0/G1 and S cell cycle phases. We expected that the modulation of NFΚB activity in PCa cells would trigger a response in the cells, due to the fact that NFΚB is reported to be activated in PCa cells [34]. The different responses of androgen-dependent and -independent cells to blocking the NFΚB pathway might be explained by different migration and invasiveness of LNCaP and DU-145 cells in which NFΚB plays a crucial role. Overall, our results show that both ERβ and NFΚB play a crucial role in the response of PCa cells to the induction of oxidative stress, and shed new light on the molecular mechanism of ZEA-induced oxidative stress in humans (Figure 6).

## 4. Conclusions

To the best of our knowledge, this is the first study to show that ZEA induces oxidative stress in PCa cell lines with different androgen sensitivities. ROS generation was observed with DNA damage and G2/M cell cycle arrest. However, the observed different sensitivities and responses to the mRNA level the of LNCaP and DU-145 cell lines indicates that the androgen-sensitivity, differentiation and metastatic potential of cells might play a role in the response of cells to ZEA. In both cell lines, the lack of active ERβ and NFΚB sensitized cells to ZEA-induced oxidative stress, suggesting that the expression of ERβ and NFΚB in PCa cells might play a protective role in ZEA exposure in hormone-dependent cancers, although this statements needs to be confirmed in further studies.

## 5. Materials and Methods

### 5.1. Cell Culture

The androgen-dependent PCa LNCaP cell line was provided by the European Collection of Authenticated Cell Cultures (EACC) (Sigma-Aldrich, Saint Louis, MO, USA), whereas the androgen-independent DU-145 cell line was purchased from American Type Culture Collection (ATCC). Cells were cultured under standard conditions in RMPI 1640 and DMEM medium, respectively (Thermo Fisher Scientific Inc/Life technologies, Waltham, MA, USA). Full growth medium was supplemented with 10% heat-inactivated Fetal Bovine Serum (FBS, 1 mM Sodium Pyruvate, 10 mM HEPES buffer and antibiotics (Penicillin 50 U/mL; Streptomycin 50 μg/mL; Neomycin 100 μg/mL). All media and supplements were purchased from Thermo Fisher Scientific Inc/Life technologies, Waltham, MA, USA. An experimental medium was used, i.e., medium serum-deprived without phenol red and antibiotics. Based on our research, the reduction of serum in the medium has no effect on PCa cell viability and proliferation.

ZEA stock solution (0.01M, Sigma-Aldrich, Saint Louis, MO, USA), 2-Phenyl-3-(4-hydroxyphenyl)-5,7-bis (trifluoromethyl)-pyrazolo stock solution [1,5-α]pyrimidine (PHTPP) (1mM, Santa Cruz Biotechnology, Dallas, TX, USA) and (E)-3-(4-t-Butylphenylsulfonyl)-2-propenenitrile stock solution (BAY 117082) (1M, Sigma-Aldrich, Saint Louis, MO, USA) were prepared in methanol and dimethyl sulfoxide (DMSO), respectively. Final ZEA, PHTPP and BAY 117082 concentrations were obtained by dilution in the experimental medium. In all experiments, cells were treated with ZEA (30 µM), ZEA (30 µM) + PHTPP (100 nM), ZEA (30 µM) + BAY (5 µM), ZEA (30 µM) + PHTPP (100 nM) + BAY (5 µM) for 48 h. The concentration of 30 µM of ZEA was chosen on the basis of our previous results [12] and a literature survey. The final concentrations of methanol and DMSO were less than 0.01%; thus, in all experiments as a control (Cnt), cells treated with the experimental medium were used. Cells treated with PHTPP (Cnt PHTPP), BAY (Cnt BAY) and both (Cnt PHTPP BAY) were also used to compare the effects of inhibitors alone.

### 5.2. Cell Viability

Cell viability was evaluated with MTT cell viability reagent (Thermo Fisher Scientific Inc/ Life technologies, Saint Louis, MO, USA). Cells were seeded at a density of 10–20 × 10^3^ cells (depending on cell line) /well on 96-well plates, and one day after seeding, the experiment was conducted. Ten microliters of MTT reagent dissolved in PBS (5mg/mL) was added to the wells after 20 h, which were then incubated for an additional four hours. Absorbance was measured at 570 nm with EL808IU BioTek microplate reader (BioTek Instruments, Inc., Winooski, VT, USA). Cell viability was expressed as % of Cnt cells (GraphPad Software, La Jolla, CA, USA, version 5). The results are presented as the mean ± SE of no less than four replicates.

### 5.3. Oxidative Stress

The number of ROS positive cells was stained with Muse Oxidative Stress Kit (Merck Millipore, Burlington, MA, USA) and counted on Muse Cell Analyzer. Cells (1 × 10^6^) were seeded on six-well plates and cultured under standard conditions. The next day, the medium was exchanged for the experimental medium, and cells were incubated for 48 h. Staining was conducted according to the manufacturer’s recommendation. The experiment was conducted in triplicate.

### 5.4. Cell Cycle

The percentage of cells in the G0/G1, S and G2 phases of the cell cycle was determined with the Muse Cell Cycle Assay Kit (Merck Millipore, Burlington, MA, USA). Cells (1 × 10^6^) were seeded and cultured as described in a previous experiment. A Cell Cycle Assay Kit was used according to the manufacturer’s recommendations. The experiment was conducted in triplicate.

### 5.5. DNA Damage

DNA damage was examined using a Muse Multicolor DNA Damage Kit (Merck Millipore, Burlington, MA, USA), according to manufacturer’s recommendations. Cells were seeded and cultured similarly to in previous experiments. Analyses were conducted on a Muse Cell Analyzer. The results of three independent experiments were expressed as % of Cnt cells.

### 5.6. Cell Nuclear Morphology—DAPI Staining

The nuclear morphology of cells was analyzed with a fluorescence microscope. Cells (15 × 10^3^) were seeded on a 96-well plate and cultured under standard conditions. The next day, the medium was exchanged for the experimental medium, and the experiment was conducted for 48 h. Then, the cells were fixed with 4% paraformaldehyde (PFA; Sigma-Aldrich, Saint Louis, MO, USA) and washed three times with phosphate- buffered saline (PBS). Then, the cells were incubated with 1μg/mL 4′,6-diamidino-2-phenylindole (DAPI; Sigma-Aldrich) before washing two times with PBS. Images were obtained using a FLoid Cell Imaging Station (ThermoFisher Scientific, Waltham, MA, USA) with an optical magnification 460×. Nuclei with nonround shape and bright blue staining represent damaged DNA.

### 5.7. Real Time qPCR (RTqPCR)

Cells were seeded on 60 mm Petri dishes and incubated to reach 90% confluency. Then cells were treated with experimental media for 48 h. Following this, total RNA was isolated with TRIzol reagent. The RNA quality and concentrations were measured on BioDrop DUO (Biodrop, Cambridge, UK). cDNA was synthesized from 5 μg of total RNA using ImProm RT-IITM reverse transcriptase (Promega, Madison, WI, USA). A RT-qPCR reaction was performed with LightCycler 96 (Roche, Basel, Switzerland) with 2 μL of cDNA. Primers were designed using Primer-BLAST (National Institute of Health, Gaithersburg, MD, USA) (Table 3). A DFS-Taq DNA Polymerase kit (BIORON, Römerberg, Germany) was used to perform the analysis. As a calibrator was used, the Human Reference RNA (Stratagene, San Diego, CA, USA) Ribosomal protein S17 (RPS17), ribosomal protein P0 (RPLP0) and histone H3.3A (H3F3A) were used as reference genes for normalization. Meting curve analysis was performed to confirm the specificity of each primer set. The qPCR array data was analyzed using the ΔΔCt method. The experiment was conducted in duplicate from three repeats.

### 5.8. Western Blot

The cells (1 × 10^6^) were seeded on Petri dishes, incubated to reach 90% confluency and induced as described previously. Protein isolation, electrophoresis, transfer and Western blot procedure were conducted as described previously [12]. Primary antibodies were used, i.e., anti-Akt (1:1000 in 5% BSA, Cell Signaling), anti-p44/42 (1:1000 in 5% BSA, Cell Signaling, Danvers, MA, USA), anti-SOD-1 (1:200, Cell Signaling, Danvers, MA, USA) or anti-GAPDH (1:1000, SantaCruz Biotechnology, Dallas, TX, USA), as a reference. Bands were visualized with the Novex^®^ AP Chromogenic Substrate (BCIP/NBT) (Thermo Fisher Scientific, Waltham, MA, USA). A densitometric analysis was conducted in ImageJ (Wayne Rasband, National Institutes of Health, Bethesda, MD, USA). The experiment was conducted in triplicate.

### 5.9. Statistical Analysis

One-way ANOVA with a Bonferroni post hoc test was used to analyze the results (GraphPad Software version 5, La Jolla, CA, USA). *p* values below 0.05 were considered statistically significant.

## Figures and Tables

**Table 1:** Relative expression of SOD-1 on protein level. The expression was calculated as a ratio of optical density of Western blot bands of SOD-1 and GAPDH. The results are expressed as fold change. ZEA—zearalenone, PHTPP—ERβ inhibitor, BAY—NFΚB inhibitor.

Treatment	Relative Expression of SOD-1 [Fold Change]
**LNCaP**	
30 µM ZEA	0.82
30 µM ZEA + PHTPP	0.80
30 µM ZEA + BAY	0.92
30 µM ZEA + PHTPP + BAY	0.99
Cnt	1.00
Cnt + PHTPP	0.96
Cnt + BAY	0.93
Cnt + PHTPP + BAY	0.80
**DU-145**	
30 µM ZEA	0.75
30 µM ZEA + PHTPP	0.91
30 µM ZEA + BAY	0.63
30 µM ZEA + PHTPP + BAY	1.32
Cnt	1.00
Cnt + PHTPP	0.84
Cnt + BAY	0.59
Cnt + PHTPP + BAY	0.57

**Table 2:** Relative expression of Akt and p44–42 on protein level. The expression was calculated as a ratio of optical density of Western blot bands. GAPDH was used as a reference gene. The results are expressed as fold change. ZEA—zearalenone, PHTPP—ERβ inhibitor, BAY—NFΚB inhibitor, n.d.—not detectable.

Treatment	Relative Expression of Akt [Fold Change]	Relative Expression of p-Akt [Fold Change]	Relative Expression of p44–42 [Fold Change]	Relative Expression of p-p44–42 [Fold Change]
**LNCaP**				
30 µM ZEA	1.09	0.99	1.03	0.89
30 µM ZEA + PHTPP	1.11	1.43	1.05	0.99
30 µM ZEA + BAY	1.35	1.66	1.03	0.94
30 µM ZEA + PHTPP + BAY	0.94	1.21	1.00	0.94
Cnt	1.00	1.00	1.00	1.00
Cnt + PHTPP	1.18	1.48	1.02	1.00
Cnt + BAY	1.08	1.12	1.04	0.94
Cnt + PHTPP + BAY	1.19	1.10	1.00	0.65
**DU-145**				
30 µM ZEA	0.75	n.d.	1.03	0.72
30 µM ZEA + PHTPP	0.91	n.d.	1.03	0.65
30 µM ZEA + BAY	0.63	n.d.	0.91	1.26
30 µM ZEA + PHTPP + BAY	1.32	n.d.	0.91	1.07
Cnt	1.00	n.d.	1.00	1.00
Cnt + PHTPP	0.84	n.d.	1.04	0.94
Cnt +++ BAY	0.59	n.d.	0.95	0.97
Cnt + PHTPP + BAY	0.57	n.d.	1.20	0.73

**Table 3:** Primers sequences. *HIF-1α*—hypoxia inducible factor 1 alpha, *SOD-1*—superoxide dismutase 1, *CDKN1A*—cyclin dependent kinase inhibitor 1A, *CDC2*—cyclin-dependent kinase 1, *NRF2*—nuclear factor erythroid 2-related factor 2, *HMOX1*—heme oxygenase 1, *IKKβ1*—inhibitor of nuclear factor kappa B kinase subunit beta, *RPS17*—ribosomal protein S17, *RPLP0*—ribosomal protein P0, *H3F3A*—histone H3.3A, bp—base pair.

Gene	Sequence (5′–3′)	Product Size [bp]
*HIF-1α*	TTACTCATCCATGTGACCATGA AGTTCTTCCTCGGCTAGTTAG	140
*SOD-1*	GCGTGGCCTAGCGAGTTAT ACACCTTCACTGGTCCATTACT	114
*CDKN1A*	GACAGATTTCTACCACTCCAA CTGAGACTAAGGCAGAAGAGT	134
*CDC2*	TTTTCAGAGCTTTGGGCACT AGGCTTCCTGGTTTCCATTT	100
*NRF2*	GTCACATCGAGAGCCCAGTC ACCATGGTAGTCTCAACCAGC	193
*HMOX1*	CAGCTCCTGCAACTCCTCAAA TTCTTCACCTTCCCCAACATTG	165
*IKKβ*	ATCCCCGATAAGCCTGCCA CTTGGGCTCTTGAAGGATACA	171
*RPS17*	AAGCGCGTGTGCGAGGAGATCG TCGCTTCATCAGATGCGTGACATAACCTG	87
*RPLP0*	ACGGATTACACCTTCCCACTTGCTAAAAGGTC AGCCACAAAGGCAGATGGATCAGCCAAG	69
*H3F3A*	AGGACTTTAAAAGATCTGCGCTTCCAGAG ACCAGATAGGCCTCACTTGCCTCCTGC	74

## Abbreviations

NFΚB	Nuclear factor kappa-light-chain-enhancer of activated B cells
PCa	Prostate cancer
ZEA	zearalenone
ERs	Estrogen receptors
ROS	Reactive oxygen species
HIF-1α	Hypoxia inducible factor 1 alpha
ERβ	Estrogen receptor β
AR	Androgen receptor
PHTPP	2-Phenyl-3-(4-hydroxyphenyl)-5,7-bis(trifluoromethyl)-pyrazolo[1,5-a] pyrimidine
EACC	European Collection of Authenticated Cell Cultures
ATCC	American Type Culture Collection
FBS	Fetal Bovine Serum
PFA	Paraformaldehyde
SOD1	Superoxide dismutase 1
CDKN1A	Cyclin dependent kinase inhibitor 1A
CDC2	Cyclin-dependent kinase 1
NRF2	Nuclear factor erythroid 2-related factor 2
HMOX1	Heme oxygenase 1
*IKKβ1*	Inhibitor of nuclear factor kappa B kinase subunit beta
RPS17	Ribosomal protein S17
RPLP0	Ribosomal protein P0
H3F3A	Histone H3.3A
